# A Practical Test in the Treatment of Pulmonary Phthisis by Gaseous Enemata

**Published:** 1887-06

**Authors:** C. A. Parker

**Affiliations:** Fort Worth, Texas


DANIEL'S
### Texas Medical Journal.

PUBLISHED MONTHLY.—SUBSCRIPTION $2.00 A YEAR.

Vol. 2. AUSTIN, JUNE, 1887. No. 12.

### ORIGINAL ARTICLES.

CONTRIBUTED EXCLUSIVELY TO THIS JOURNAL.

*The Articles in this Department are accepted and published with the understanding that we are not responsible for, nor do we indorse the views and opinions of the writers by so doing.*

### A PRACTICAL TEST IN THE TREATMENT OF PULMONARY PHTHISIS BY GASEOUS ENEMATA.

*By C. A. Parker, M. D., Fort Worth, Texas.*

For Daniel's Texas Medical Journal.

MY experience, including a period of forty days with the above remedy, has been most gratifying. I can say to a positive certainty, that this treatment has done more in my hands so far, than any other method yet employed, though my knowledge of its curative powers, is yet in its infancy. I have used this treatment with six cases,—four of Phthisis Pulmonalis, and one of Asthma, four of which I most respectfully submit:

CASE I. Miss B. aged 20, by occupation a seamstress, rather stooped, weight 130 pounds in health, (A father, brother and sister died with Phthisis.) Applied to me for treatment, April 27. She had been treated by several physicians previously, with the usual remedies, given for this most formidable disease. Upon examination, I found vesicular respiration almost extinct, over entire right lung; also upper lobe of left involved to an extent seemingly beyond a hope of recovery; rapid respiration, increased hearts action, disturbed sleep, an abnomal craving appetite, very profuse night sweats, with œdema of lower extremeties. She suffered continually from indigestion to such an extent, that any amount of food taken in the stomach produced great pain and nausea; Temperature ranged from 102 to 103½. Every afternoon the smallest amount of exertion produced great fatigue and intense fits of coughing.

I advised her, to take the gaseous treatment, to which she readily consented.

After first administration she seemed to be aroused; a feeling of bouyancy, or well being, and she expressed herself as feeling better than she had for several months past. She slept the following night, but had profuse sweats, which ceased after the second enema.

I continued the treatment daily, and in ten days she was much improved; had gained from twenty-eight to forty-two ounces each week. All swellings left the extremities; night sweats ceased and she gained strength very rapidly; digestion became as it was before she had the decline of health; cough which was indeed very annoying and troublesome, almost entirely cured; expectoration was reduced from thirty ounces each day, to three or less, in same length of time. She is now able to dress, and take her daily walks with ease and very little fatigue.

CASE 3.—John L., received May 10th, age 29; occupation, an engineer; father died of consumption at 39 years of age. An examination revealed a temperature of 103½ F., respirations forty per minute; pulse rapid; night sweats profuse; cough continual, with diarrhœa. The right and a portion of left lung involved; rapid emaciation with great weakness. I diagnosed this as a case of incipient phthisis, very rapid in its course.

From first injection he had all these symptoms much improved; has gained forty ounces each week and now does all necessary work as an office boy.

CASE 4. Mr. M. A. A., Age 32, has had cough for five years. A native of N. Y. State, recv'd May 24th, very much emaciated, cough incessant; night sweats with great weakness. Temperature 104 at evening. After each enema he expressed himself as feeling very much better, and after four injections ceased to visit the office and returned to manual labor, thinking he was cured; but I am informed, he is now growing worse, since he ceased to take treatment and will return soon again to resume treatment. I hope to refer to him again.

CASE 6. Miss Belle G. Age 32, receiv'd., May 25th, suffering from an attack of asthma. (chronic) Two enemas cured her of the present attack and she has not had another symptom since.

The other three cases are the same relatively, to the ones just reported.

I will state, that these cases have all gone through the long list of usual remedies, before applying to me for treatment. All these cases with several new ones, will be reported better, after an other months treatment, the agent proving most satisfactory to myself and patients.

After trying Blue Lick water, I used thirty-six grains of Pot. Sulphuret, disolved in thirty-two ounces of water; through this a stream of Carb. Acid gas is passed (produced by the action of Sulphuric Acid on Bicarb Sod.) in to the bowels, on an average about three quarts, at each sitting, consuming about fifty minutes of time. My experience teaches me, that any thing more diluted is not efficient as the success depends on the amount, and the time consumed in giving. I have had no unpleasant results in a single case yet, except a transient pain produced by detension, which passes very rapidly away, leaving the patient cheerful and bright. The time for using this has been two hours after meals, once daily till patient begins to improve; these as circumstance demand, but I am inclined to the opinion that one hour after meals is better, because of its stimulating effects on the entire system; hence its aid in digestion. I never use gas over two hours old.

This method has done more in my hands than any thing else I have yet known. Its effects are somewhat surprising I must confess, and hence this is now my conclusion until I am convinced otherwise. The remedy is antiseptic, and a germicide by the law of exos and endosmosis, because of its rapid elimintion and immediate results.

This concludes the present report, but I hope to hear from others on this important subject through the medium of the JOURNAL.

